# Parental Exposure to Dim Light at Night Prior to Mating Alters Offspring Adaptive Immunity

**DOI:** 10.1038/srep45497

**Published:** 2017-03-31

**Authors:** Yasmine M. Cissé, Kathryn L.G. Russart, Randy J. Nelson

**Affiliations:** 1 Department of Neuroscience, Neuroscience Research Institute, Behavioral Neuroendocrinology Group, The Ohio State University Wexner Medical Center, Columbus, OH 43210, USA

## Abstract

Exposure to dim light at night (dLAN) disrupts natural light/dark cycles and impairs endogenous circadian rhythms necessary to maintain optimal biological function, including the endocrine and immune systems. We have previously demonstrated that white dLAN compromises innate and cell mediated immune responses in adult Siberian hamsters (*Phodopus sungorus*). We hypothesized that dLAN has transgenerational influences on immune function. Adult male and female Siberian hamsters were exposed to either dark nights (DARK) or dLAN (~5 lux) for 9 weeks, then paired in full factorial design, mated, and thereafter housed under dark nights. Offspring were gestated and reared in dark nights, then tested as adults for cell-mediated and humoral immunity. Maternal exposure to dLAN dampened delayed type hypersensitivity (DTH) responses in male offspring. Maternal and paternal exposure to dLAN reduced DTH responses in female offspring. IgG antibodies to a novel antigen were elevated in offspring of dams exposed to dLAN. Paternal exposure to dLAN decreased splenic endocrine receptor expression and global methylation in a parental sex-specific manner. Together, these data suggest that exposure to dLAN has transgenerational effects on endocrine-immune function that may be mediated by global alterations in the epigenetic landscape of immune tissues.

Since the turn of the 20^th^ century, the planet has undergone a marked increase in nighttime illumination. The adoption of electrical lighting has occurred without consideration or understanding for the wide-ranging physiological and psychological effects that light at night (LAN) exerts on both humans and wildlife. Artificial LAN is present in nearly all ecosystems^1^,^2^ and has wide ranging effects on fitness^3^. LAN reduces mate selectivity and alters timing of reproduction^4^,^5^,^6^. Immune responses are reduced in adult rodents exposed to LAN; specifically, cell-mediated immune responses to a chemical irritant and blood bactericidal capacity are suppressed^7^.

Optimal immune function depends on an intact circadian system^8^,^9^. In mammals, the circadian system comprises a hierarchy of biological timekeepers. The master biological clock is located in the ventral hypothalamus of the brain, within a paired structure termed the suprachiasmatic nucleus (SCN). The mechanism of the circadian clock arises from a transcriptional auto-regulatory feedback loop with a period of about 24 hours, that is generated from a defined set of core clock genes^10^. Synchronization of the SCN to the solar day is critical for proper physiological functioning^11^; the SCN is exquisitely sensitive to light information and LAN can disrupt circadian rhythms with significant downstream physiological and behavioral effects^12^. LAN likely influences immune function by disrupting circadian organization. Disruptions in circadian organization have wide ranging effects on physiology outside the immune system. Recent studies suggest that parental endocrine disruption has transgenerational effects on offspring physiology and behavior. LAN exposed animals also display wide ranging alterations in endocrine profiles^13^. The extent to which these effects on physiology and behavior persist or are transmitted across generations remains unspecified.

In the present study, we assessed dLAN mediated changes in adaptive immunity in adult offspring and demonstrate that parental exposure to dLAN prior to mating is sufficient to decrease adult offspring cell-mediated immunity and enhance humoral immune function in a parent- and offspring-sex specific manner. These changes are accompanied by altered splenic endocrine receptor expression in offspring sired by studs exposed to dLAN, relative to offspring of studs exposed to dark nights, suggesting altered neuroendocrine-immune communication. To address these transgenerational effects in immune function, we evaluated splenic methylation status and determined that parental exposure to dLAN decreased global splenic methylation. These results indicate pervasive and transgenerational effects of light at night on the immune system.

## Results

### Exposure to dLAN Increased Body Mass in Parents but Not Offspring

The present study exposed adult Siberian hamsters to 9 weeks of nightly dim (5 lux) white light (dLAN) or a standard light/dark (DARK) cycle. Consistent with previous reports 14 exposure to nine weeks of dLAN increased body mass in adult male and female Siberian hamsters (*Phodopus sungorus*) (F_1,42_ = 6, *p* < 0.05; Table 1). Overall, males weighed more than females (F_1,42_ = 32, *p* < 0.001).

**Figure 1:**
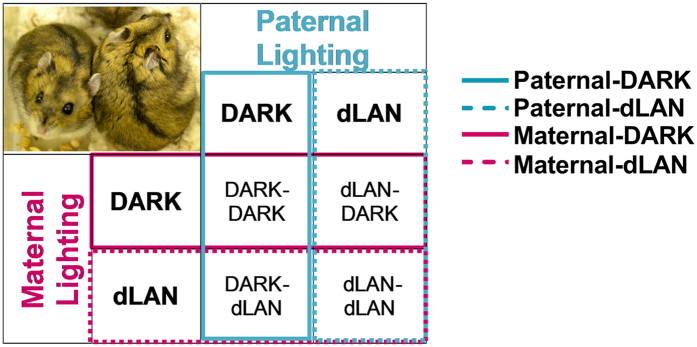
DARK represents parents housed in a standard 16 h: 8 h light/dark cycle (150 lux: 0 lux). dLAN represents parents housed in 16 h: 8 h light/dim cycle (150 lux: 5 lux). DARK- DARK (Male-Female; n = 5), DARK-dLAN (n = 6), dLAN-DARK (n = 5), and dLAN-dLAN (n = 7). Blue boxes represent paternal lighting groups: dark (solid) and dLAN (dashed). Pink boxes represent maternal lighting conditions: dark (solid) and dLAN (dashed).

Parents were paired in a full factorial fashion described in Fig. 1. Briefly, this pairing resulted in four groups described as father’s lighting conditions – mother’s lighting condition: DARK-DARK, DARK-dLAN, dLAN-DARK, and dLAN-dLAN. All animals were paired, mated, and transferred into standard light conditions with dark nights where they remained thereafter. All pups were thus gestated, born, and maintained in standard light-dark conditions. Parental exposure to dLAN did not affect offspring body mass (*p* > 0.05; Table 1). At 8 weeks of age male offspring weighed more than female offspring (F_1,80_ = 28, *p* < 0.001).

### Parental Exposure to dLAN Dampened Delayed Type Hypersensitivity in a Parent and Offspring Sex-Specific Manner

**Figure 2:**
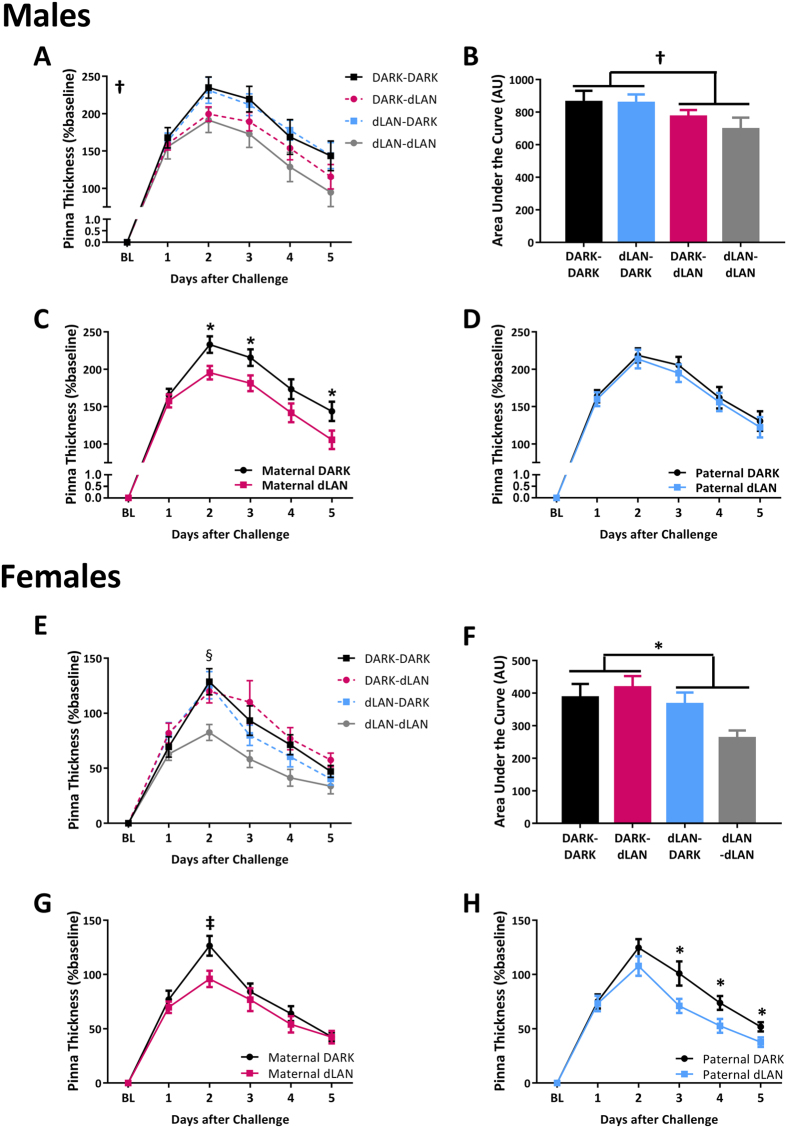
Maternal exposure to dLAN dampens male offspring cell-mediated immune responses (**A**–**D**). Paternal exposure to dLAN dampens female offspring cell-mediated responses (**E**–**H**). Percent baseline swelling of pinna in offspring based on parental lighting condition (father-mother; **A**,**E**) and area under the curve of swelling response (**B**,**F**). Percent baseline swelling of pinna when grouped by maternal (**C**,**G**) or paternal (**D**,**H**) lighting condition. n = 5–14 per group per sex. Data presented as mean ± SEM. ^†^*p* < 0.05 maternal exposure to dLAN vs DARK; *p ≤ 0.05 dLAN vs DARK; ^‡^*p* < 0.05 paternal exposure to dLAN vs DARK; ^§^*p* < 0.05 dLAN-DARK vs dLAN-dLAN.

To assess cell-mediated immune function, we examined delayed type hypersensitivity (DTH) responses in adult offspring. DTH is an antigen specific T-cell mediated immune response, considered an index of effector T cell function and requires specific antigen memory^15^. Inflammation occurs at the site of a second antigenic challenge, reproduced in the lab by application of 2–4-dinitro-1-fluorobenzene (DNFB), as a result of invading monocytes and lymphocytes infiltrating the dermis and epidermis^16^. This swelling reaction is positively correlated with T-cell mediated immune response. DNFB-challenge induced a swelling reaction in the left pinna of all experimental groups (F_4,289_ = 236, *p* < 0.001; Fig. 2A,E). Males displayed a more pronounced swelling reaction to antigenic challenge (F_4,289_ = 29, *p* < 0.001), as a result males and females were analyzed separately.

#### Male Offspring

As compared to male offspring of dams maintained under dark nights, male offspring of dams exposed to dLAN displayed reduced pinna swelling over time in response to a secondary DNFB challenge as determined by area under the curve (F_1,48_ = 4, *p* < 0.05; Fig. 2B). Specifically, sons of dams exposed to dLAN reduced swelling 2, 3, and 5 days post-challenge (F_1,47_ = 6, *p* < 0.05, F_1,47_ = 5, *p* < 0.05, and F_1,47_ = 4, *p* < 0.05, respectively; Fig. 2C).

#### Female Offspring

Female offspring of studs exposed to dLAN displayed decreased pinna swelling over time to a secondary DNFB challenge, as determined by area under the curve (F_1,29_ = 5, *p* < 0.05; Fig. 2F). Maternal exposure to dLAN decreased female offspring swelling response only on day 2 after challenge (F_1,29_ = 4, *p* = 0.05; Fig. 2G). Paternal exposure to dLAN reduced swelling response in female offspring 3, 4, and 5 days post-challenge (F_1,29_ = 7, *p* < 0.05, F_1,29_ = 5, *p* < 0.05, and F_1,29_ = 5, *p* < 0.05, respectively; Fig. 2H).

### Maternal Exposure to dLAN Enhanced Antibody Production to a Novel Antigen

**Figure 3:**
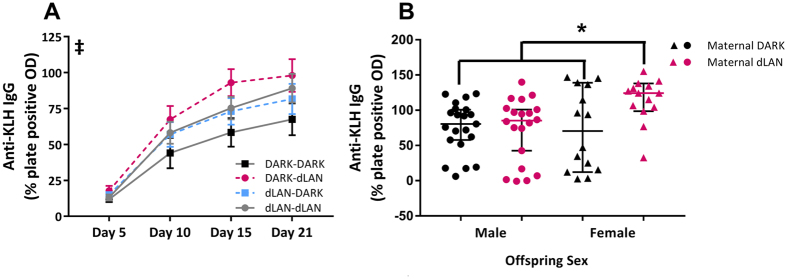
Maternal exposure to dLAN enhances offspring humoral immunity. Anti-Keyhole Limpet Hemocyanin IgG titers in offspring (**A**). At 21 days post-injection, anti-KLH IgG titers in offspring based on maternal lighting condition (**B**). n = 13–20 per group. Data presented as mean ± SEM. Non-parametric data are presented as median with 95% confidence interval. ^‡^*p* < 0.05 maternal dLAN vs DARK. *p < 0.05 female offspring of maternal dLAN vs maternal-DARK males and females and maternal-dLAN males.

Four months following DTH testing, hamsters were tested for antibody response to a presumably novel antigen, keyhole limpet hemocyanin (KLH), a respiratory protein of giant keyhole limpet. KLH produces an antigenic response without a febrile response^17^. Titers of anti-KLH antibodies serve as a measure of humoral immune response. Exposure to KLH induced production of anti-KLH IgG production over time (F_2,107_ = 157.14, *p* < 0.001). Maternal exposure to dLAN enhanced production of anti-KLH IgG over time (F_2,107_ = 3.19, *p* < 0.05; Fig. 3A). Paternal exposure to dLAN did not affect antibody production (*p* > 0.05). Offspring sex and maternal lighting condition interacted at 21 days post-injection (X^2^ = 11.82, *p* < 0.01; Fig. 3B), such that female offspring of dLAN-mothers increased anti-KLH IgG relative to both males and females of DARK-mothers (Dunn’s, *p* < 0.05).

### Paternal Exposure to dLAN Alters Offspring Splenic Melatonin (MT1) and Glucocorticoid Receptor (GR) Expression

**Figure 4:**
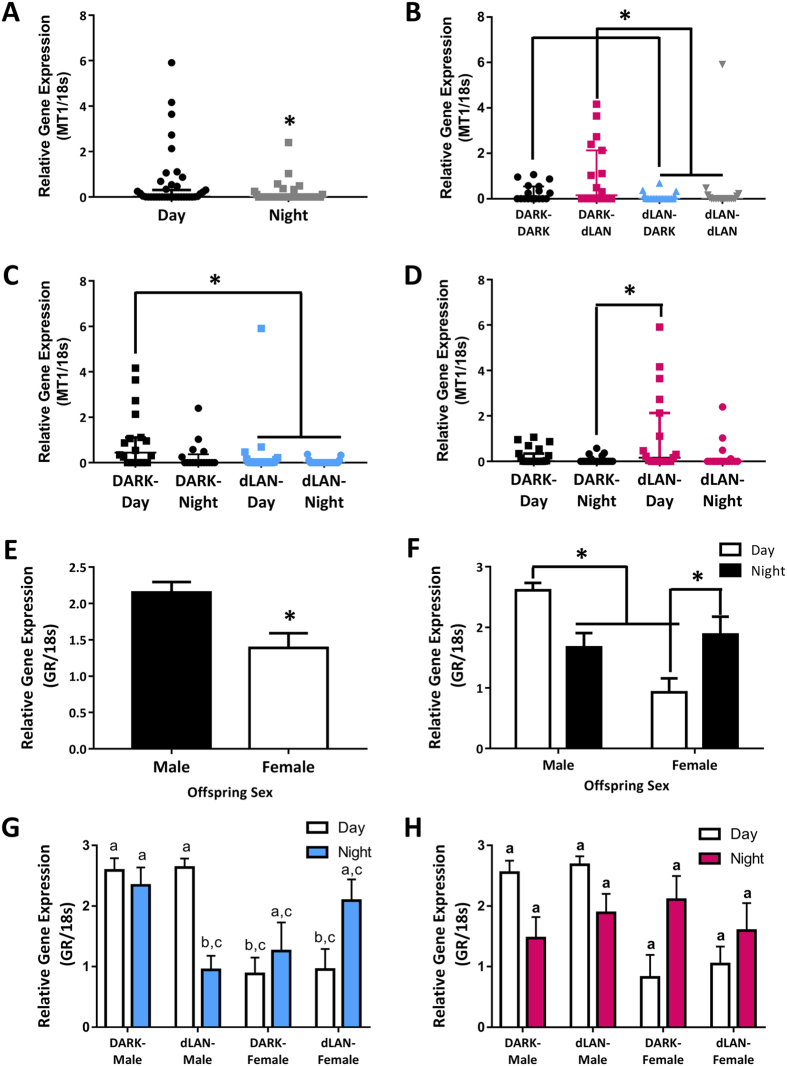
Paternal exposure to dLAN alters splenic MT1 and GR expression. Splenic MT1 expression by time of day (**A**), parental (**B**), paternal (**C**), and maternal lighting condition (**D**). Splenic GR expression by offspring sex (**E**), offspring sex by time of day (**F**), offspring sex by time of day by paternal lighting (**G**), offspring sex by time of day by maternal lighting (**H**). Parametric data presented as mean ± SEM. Non-parametric data are presented as median with 95% confidence interval. n = 4–14 per group per sex. *p < 0.05. Different letters indicate significant differences between groups.

Exposure to light at night alters endocrine function^13^, and elimination of nightly melatonin secretion is the most pronounced endocrine effect of light at night. Melatonin is a potent immunomodulator and enhances immune responses by stimulating bone marrow proliferation, antigen presentation, and release of certain cytokines, namely IL-2 and IFN-γ^18^,^19^,^20^. The majority of immune cells and organs express melatonin receptors. Melatonin increases MT1 activity in the thymus and the spleen and enhances DTH response in Syrian hamsters^21^. We therefore evaluated MT1 as a potential mediator of decreased adaptive immune response in offspring. Splenic MT1 expression varied over the course of the day such that MT1 expression was increased during the day relative to nighttime (U = 533.00, *p* < 0.05; Fig. 4A). Paternal exposure to dLAN decreased splenic MT1 expression in offspring relative to their counterparts, whose fathers were housed in dark nights (U = 345.00, *p* < 0.001; Fig. 4C). Maternal exposure to dLAN increased daytime MT1 expression relative to offspring of DARK-mothers at nighttime (X^2^ = 8.01, *p* < 0.05; Fig. 4D). Maternal and paternal lighting conditions interacted (U = 19.16, *p* < 0.001; Fig. 4B), such that offspring of dLAN-DARK and dLAN-dLAN parentage expressed less than those with only a mother in dLAN (DARK-dLAN) (Dunn’s, *p* < 0.05).

Interactions of the endocrine and immune systems are crucial to proper immune function^22^. Glucocorticoids suppress immune responses in order to prevent runaway inflammation, but excess glucocorticoid production can impair immune responses^23^. Chronic elevation of cortisol upregulates expression of the glucocorticoid receptor (GR) on splenic cells^24^. Long-term glucocorticoid signaling in the spleen was evaluated through glucocorticoid receptor gene expression as a mediator of altered immune responsiveness in offspring. Males had higher splenic GR expression than females (F_1,68_ = 14, *p* < 0.001; Fig. 4E). There was an interaction between sex and time of day (F_1,68_ = 14.95, *p* < 0.05; Fig. 4F) such that males decreased splenic GR at night, whereas females increased GR expression at night (Tukey’s, *p* < 0.01). Paternal lighting condition interacted with offspring sex and time of day (F_1,68_ = 5.55, *p* < 0.05; Fig. 4G), such that paternal exposure to dLAN decreased GR expression in male offspring at night (Tukey’s, *p* < 0.05), but did not affect female offspring (*p* > 0.05). Maternal exposure to dLAN did not affect offspring splenic GR expression (*p* > 0.05; Fig. 4H).

### Parental Exposure to dLAN Alters Splenic Methylation

**Figure 5:**
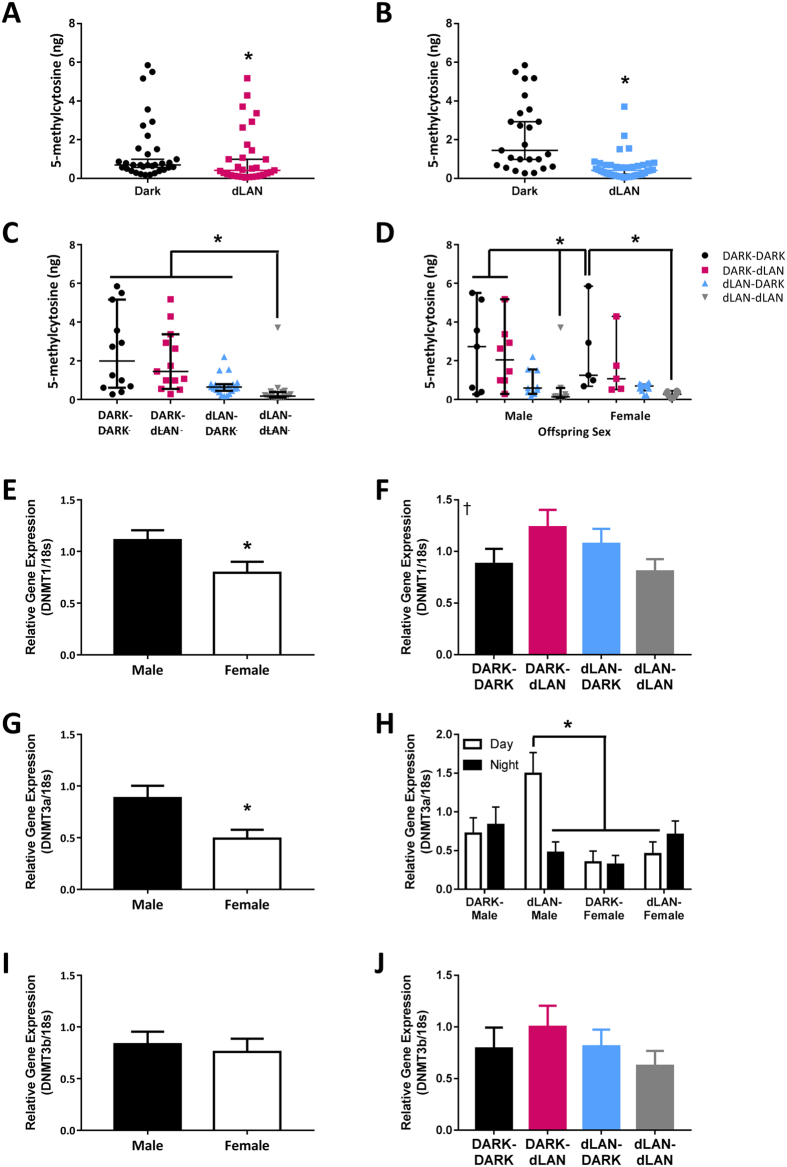
Parental exposure to dLAN decreases global splenic methylation. Absolute splenic methylation by maternal (**A**), paternal (**B**), parental (**C**), and offspring sex by parental lighting condition (**D**). Relative gene expression of DNMT1 by sex (**E**), and parental light condition (**F**). Relative gene expression of DNMT3a by sex (**G**), and offspring sex by paternal lighting condition by time of day (**H**). Relative gene expression of DNMT3b by sex (I), and parental light condition (J). Parametric data presented as mean ± SEM. Non-parametric data are presented as median with 95% confidence interval. n = 5–12 per group per sex. *p < 0.05; ^†^*p* < 0.05 interaction of maternal and paternal lighting condition.

Global splenic methylation was assessed using a methylated DNA ELISA to investigate the role of CpG methylation in the inheritance of the transgenerational immune phenotype. Maternal and paternal (U = 360.00, *p* < 0.05, and U = 155.00, *p* < 0.001, respectively; Fig. 5A and B) exposure to dLAN decreased methylation in the spleen. There was an interaction between maternal and paternal lighting conditions (X^2^ = 29.39, *p* < 0.001) such that dLAN/dLAN offspring had less splenic methylation than any other group (Dunn’s, *p* < 0.05; Fig. 5C). Additionally, there was an interaction between paternal and maternal lighting conditions and offspring sex (X^2^ = 29.86, *p* < 0.001; Fig. 5D) such that dLAN-dLAN males reduced splenic methylation relative to DARK-DARK and DARK-dLAN male offspring. DARK-DARK females had higher splenic methylation than dLAN-dLAN females and dLAN-dLAN male offspring (Dunn’s, *p* < 0.05).

Alterations in DNA methyltransferase (DNMT) gene expression were investigated as a possible influence on changes in splenic methylation state. DNMT1 is responsible for maintaining DNA methylation at hemimethylated sites, whereas DNMT3a and 3b catalyze *de novo* methylation^25^,^26^. Male offspring expressed increased DNMT1 and DNMT3a in the spleen relative to their female counterparts (F_1,71_ = 5.00, *p* < 0.05 and F_1,62_ = 6.96, *p* < 0.05: Fig. 5E and G respectively). Maternal and paternal lighting conditions interacted in offspring splenic DNMT1 expression (F_1,71_ = 4.84, *p* < 0.05, Fig. 5F), but post hoc testing was unable to detect the source of these significant interactions. Paternal lighting condition, offspring sex, and time of day interacted for DNMT3a (F_1,62_ = 5.23, *p* < 0.05; Fig 5H) such that male offspring from dLAN fathers had greater expression of DNMT3a during the day than both their counterparts at night and female offspring (Tukey’s, *p* < 0.05). DNMT3b expression was not altered by offspring sex, parental lighting, or time of day (p > 0.05; Fig 5I and J).

## Discussion

Long-term parental exposure to dim light at night prior to breeding was sufficient to depress cell-mediated immunity in offspring and enhance humoral immunity in a parent and offspring sex-specific manner (Figs 2, 3). Expression of endocrine receptors, namely melatonin and glucocorticoid receptor, were decreased in the spleens of offspring whose fathers were exposed to dLAN (Fig. 4). Both maternal and paternal exposure to dLAN decreased global methylation in the spleens of their offspring, but this decrease was not observed in DNMTs, suggesting that there may be additional epigenetic mechanisms such as histone modification at play to generate this offspring phenotype (Fig. 5). Parental dLAN exposure prior to conception is sufficient to alter offspring immunological responses and epigenetic landscape. These data demonstrate that a seemingly innocuous stimulus, dim light exposure at night, is sufficient to induce transgenerational effects on physiology.

In common with adult exposure to dLAN^27^, offspring of parents exposed to dLAN displayed decreased cell-mediated immunity (Fig. 2). Swelling reactions to DNFB are due to leukocyte infiltration into the dermis and epidermis, and the magnitude of swelling is positively correlated with the intensity of immune reaction^15^. Swelling reactions in offspring were equivalent at 24 hours, but were significantly depressed beginning at 48 hours (Fig. 2A and E). DTH responses in offspring from parents exposed to dLAN only begin to diverge from those with parents exposed to dark nights at Day 2. The timing suggests that initial infiltration by macrophages and neutrophils was not altered by parental exposure to dLAN, but instead the secondary recruitment or polarization of helper T-cells may be impaired^28^,^29^.

T-cell polarization and cell-mediated immune responses are sensitive to hormonal influences. Therefore, we evaluated MT1 and GR expression to investigate immune-endocrine signaling in the spleens of offspring. In addition to their effects on the immune system, glucocorticoids and melatonin regulate endocrine receptor expression in immune tissues. Elevated physiological cortisol concentrations upregulate expression of its low affinity receptor (GR) on splenic cells, while simultaneously downregulating melatonin receptors (MT1 and MT2)^24^. Melatonin, conversely, downregulates splenic GR expression^30^ and upregulates expression of both melatonin receptors^24^. Decreased expression of both MT1 and GR in the spleens of offspring sired by males exposed to dLAN (Fig. 4) suggests a disruption in endocrine signaling. Previous reports have indicated that low levels of light at night are not stressful to adult rodents^31^, suggesting that the effects we observed were not due to prenatal stress. Here we saw opposing splenic GR expression during the day versus the night. This pattern was driven by a sharp decrease from daytime to nighttime splenic GR expression in male offspring of dLAN-fathers (Fig. 4G). However, in females the pattern is not significantly driven by dLAN, and may change under control conditions.

Melatonin is immediately diminished by exposure to light at night in both humans and rodents^32^,^33^. MT1 expression is suppressed in the brains of Siberian hamsters on the first night of constant light^34^. Interestingly, splenic MT1 expression during the night was extremely low or not present, and effects of nighttime lighting only affected daytime gene expression. Maternal dLAN increased daytime MT1 expression, inverse to the decrease observed in offspring of fathers exposed to dLAN (Fig. 4D). Offspring of dLAN-dLAN parents also decrease MT1 expression, indicating the paternal effect overrides the maternal increase. The number of offspring generated did not allow for sufficient round-the-clock sampling to determine a change in the circadian pattern of melatonin or cortisol, but given these receptor gene data, this area warrants further investigation in the future.

MT1 expression was the only factor assessed here that showed an effect of maternal exposure to dLAN, and the effect was overridden by paternal lighting condition. This may be due to the increased time that offspring spend with their mothers relative to their fathers in this design; fathers are removed after one week of pairing, whereas mothers remain with offspring until weaning. This design allows pre-natal effects of the placental environment as well as post-natal effects of maternal care and nursing. Indeed, when looking at global methylation maternal dLAN decreased global methylation but to a much lesser extent than paternal dLAN. These results suggest that mothers may be able to compensate for some of the effects of pre-natal dLAN but some deficits still remain leading to impaired immune function. Previous studies indicate many symptoms from exposure to dLAN diminish after 1 week and are eliminated by 3 weeks^35^. Therefore, dLAN effects may diminish in dams during gestation while still influencing the offspring, which may underlie the lack of maternal effects.

Global methylation assays are a method of analyzing broad changes in methylation of the genome. In this case, parental dLAN decreased global methylation of the spleen in offspring. We then assessed whether reduced DNMT expression contributed to the decreased methylation state and found that DNMT expression was relatively unaltered, if not increased. The methylation data also did not account for the sex difference in baseline DNMT1 and DNMT3a expression. Although a useful general measure, global methylation does not take into account all aspects of chromatin state. Discrepancies in methylation can also be explained by proportional differences in euchromatin versus heterochromatin or other types of histone modification. Further studies on chromatin state and HDAC expression may elucidate these differences.

Transgenerational effects on physiology and behavior are often skewed towards male offspring^36^. In the present study, immune function in male and female offspring were equally affected by parental exposure to dLAN (Figs 2, 3), but were differentially affected with regards to the genes assessed (Figs 4 and 5). Sex differences in offspring phenotype have been associated with sexual dimorphisms in the embryo prior to implantation^37^ or sex differences in placental responses to the maternal environment^38^. In both cases, the adult phenotype is a result of sex chromosome-driven alterations in gene expression, as sex hormones are not always produced during these developmental stages. The X chromosome contains genes involved in immune function, such as CD40 ligand, IL-2 receptor-γ, and IKBK-γ^39^. Disruptions in the epigenetic landscape of the genome were observed in our study (Fig. 5); it is unlikely that genes on the X chromosome are exempt from this disruption. Modulations of X-linked immune genes could play a role in the sexual dimorphism in offspring phenotype, specifically, in the parent to offspring sex specific effects of DTH responses (Fig. 2).

In conclusion, these data suggest that parental exposure to dLAN prior to mating alters adult offspring adaptive immune function. This is the first report, to our knowledge, to illustrate a transgenerational effect of exposure to light at night. Given the widespread use of electrical lighting and devices late into the night, it is critical to evaluate the potential transgenerational effects of dLAN in other physiological and behavioral systems. Altered immune responses in offspring that have only experienced dLAN prior to conception indicates that seemingly innocuous, but pervasive, nighttime lighting may have transgenerational effects on physiology.

## Methods

### Animals

#### Parents

Male (n = 23) and female (n = 23) Siberian hamsters (*Phodopus sungorus*) were obtained from our in-house breeding colony at The Ohio State University. Hamsters were maintained on a standard 16 h: 8 h light/dark cycle (DARK; 150 lux: 0 lux) with lights on at 22.00 h and off at 14.00 h EST, in polypropylene cages (30 × 15 × 14 cm) on static racks in a temperature- and humidity-controlled vivarium. All animals were provided *ad libitum* access to food (Harlan Teklad 8640; Madison, WI, USA) and filtered tap water. All experiments were approved by the Ohio State University Institutional Animals Care and Use Committee, and animals were maintained in accordance with the recommendations of the National Institutes of Health and *The Guide for the Care and Use of Laboratory Animals*.

#### Generation of F_1_

Adult (>8 week) Siberian hamsters were individually housed and randomly assigned to a lighting condition: DARK or dLAN (light (150 lux):dim (5 lux)). Hamsters were maintained in respective lighting conditions for 9 weeks at which point all animals were paired and mated as described in Fig. 1, and thereafter housed in standard DARK conditions. Pairings resulted in four parental groups: DARK-DARK (Male/Female; n = 5), DARK-dLAN (n = 6), dLAN-DARK (n = 5), and dLAN-dLAN (n = 7). Males were removed one week after pairing. Of all pairings, 5 did not successfully mate within this time window (1 DARK/DARK, 2 DARK/dLAN, and 2 dLAN/dLAN) and one litter cannibalized their pups (1 dLAN/dLAN). Remaining pups (n = 88) were weaned at 21 days of age and group-housed with same sex siblings. At 7 weeks of age hamsters were individually housed. All experimental manipulations occurred once offspring reached adulthood (>8 weeks of age).

### Delayed Type Hypersensitivity (DTH)

Hamsters were sensitized to a chemical antigenic challenge, 2-4-dinitro-1-fluorobenzene (DNFB; Sigma, St. Louis, MO). Animals were lightly anaesthetized with isoflurane vapor and an area of ~2 × 3 cm was shaved on the dorsum. Approximately 25 μL of DNFB [0.05% vol/vol in 4:1, acetone:olive oil vehicle] was applied to the shaved skin via pipette for the first two days (sensitization). Seven days later hamsters were challenged on the left pinna with 20 μL of DNFB [0.5% vol/vol in 4:1 acetone to olive oil vehicle], while the right pinna was treated with 20 μL vehicle alone. Pinna thickness was measured using a constant loading dial micrometer (Mitutoyo America, Aurora, IL) in order to determine baseline thickness and ensuing swelling. The thickness of both pinnae was measured every 24 h for the next 5 days by the same investigator (Y.M.C.) from 11.00 to 12.30 h.

### Keyhole Limpet Hemocyanin (KLH)

Hamsters were deeply anesthetized using isoflurane and blood was collected from the retro-orbital sinus into heparinized microcapillary tubes. Immediately following blood collection, hamsters were injected intraperitoneally with 150 μg of KLH (CalBiochem, LaJolla, CA) in 100 μL Freund’s incomplete adjuvant. Blood was then collected in a similar manner at 5, 10, and 15 days post injection. Blood samples were centrifuged at 4 °C, plasma was removed, and stored at −80 °C until ELISAs were performed.

### KLH ELISA

Plasma samples were thawed, diluted in PBS-Tween (1:20; Sigma, St.Louis, MI), and plated on 96- well plates coated with KLH in duplicate. Positive and negative controls, from KLH exposed and naïve hamsters respectively, were also plated in duplicate. Plates were incubated at 37 °C for 1 h and washed with PBS-Tween, before addition of alkaline phosphatase conjugated anti-mouse IgG (1:500; MP Biochemicals, Aurora, OH). Plates were incubated once again at 37 °C for 1 h, washed with PBS-Tween, then treated with *p-*nitrophenyl phosphate for 20 min and read at 405 nm on a spectrophotometer.

### Blood and Tissue Collection

Twenty-one days following KLH immunization, hamsters were anesthetized with isoflurane vapors and a blood sample was collected from the retro-orbital sinus. Hamsters were deeply anesthetized with isoflurance vapors and rapidly decapitated and tissues were removed and flash frozen on dry ice for subsequent qPCR analysis.

### Quantitative PCR (qPCR)

Total RNA from spleens was extracted using Trizol reagent (Qiagen). DNA was lysed using DNAse 1 Amplification grade (Invitrogen, Carlsbad, CA). RNA was reverse transcribed into cDNA using M-MLV Reverse Transcriptase enzyme (Invitrogen, Carlsbad, CA) according to the manufacturer’s instructions. Splenic GR, MT1, DNA Methyltransferase 1 (DNMT1), DNMT3a, and 3b expression were assessed, using primers previously described for Siberian hamster GR^40^, MT1^4^, and DNMTs 41 on an ABI 7500 Fast Real Time PCR system using SyBR Green PCR Master Mix. Cycling conditions were: 95 °C for 5 min, followed by 40 cycles of 95 °C for 15 sec and 60 °C for 1 min for GR and MT1, and 95 for 10 sec 60 C for 30 sec and 72 C for 30 sec for the DNMTs^41^.

### Methylated DNA ELISA

Total DNA from spleens was isolated using a Genomic DNA Isolation Kit (Ab65358, Abcam, Cambridge, UK). Global DNA methylation status was quantified using a Colorimetric Methylated DNA Quantification Kit (Ab117128, Abcam, Cambridge, UK) according to manufacturer instructions. Absolute quantities of methylated DNA were calculated using the provided formula 5-mC (ng) = (Sample OD- Negative Control OD)/(Slope x 2).

### Data Analyses

Statistical analyses were conducted using SPSS Statistics v 22 (IBM; Armonk, NY). Comparisons of DTH swelling and KLH IgG responses between groups were assessed using repeated measures ANOVA. Two way ANOVAs, assessing the effects of parental lighting condition, offspring sex, and interactions, were run on all other data. Post hoc tests of statistically significant groups were performed using Tukey’s HSD test. If the data did not meet the assumptions of normality or equal variance, then nonparametric statistic were conducted. Differences between means were considered statistically significant when *p* ≤ 0.05 for all analyses.

## Additional Information

**How to cite this article**: Cissé, Y. M. *et al*. Parental Exposure to Dim Light at Night Prior to Mating Alters Offspring Adaptive Immunity. *Sci. Rep.* **7**, 45497; doi: 10.1038/srep45497 (2017).

**Publisher's note:** Springer Nature remains neutral with regard to jurisdictional claims in published maps and institutional affiliations.

## Figures and Tables

**Table 1:** Body mass of parents after 9 weeks of DARK or dLAN and body mass of offspring at 8 weeks of age.

**Parents**				
Mean	DARK		dLAN	
	39.9719 g		42.3404 g*	
SD	5.35353		4.46007	

Mean	Males		Females	
	44.3643 g		38.1539 g^**†**^	
SD	3.85817		3.95206	

**Offspring**				
Mean	DARK-DARK	DARK-dLAN	dLAN-DARK	dLAN-dLAN
	37.885 g	37.3867 g	36.0446 g	35.44 g
SD	4.90364	4.67793	4.96728	3.56372

Mean	Males		Females	
	38.4993 g		33.5479 g^**†**^	
SD	3.69104		4.30158	

*p < 0.05 DARK vs dLAN; ^†^*p* < 0.05 Males vs Females.
